# Successful Community Midwives in Pakistan: An Asset-Based Approach

**DOI:** 10.1371/journal.pone.0135302

**Published:** 2015-09-02

**Authors:** Zubia Mumtaz, Adrienne V. Levay, Afshan Bhatti

**Affiliations:** 1 School of Public Health, University of Alberta, Edmonton, Alberta, Canada; 2 Real Medicine Foundation Pakistan, Islamabad, Pakistan; University of Edinburgh, UNITED KINGDOM

## Abstract

In response to the low levels of skilled birth attendance in rural Pakistan, the government introduced a new cadre of community midwives (CMWs) in 2006. Assessments to-date have found that these CMWs have yet to emerge as significant providers for a number of sociocultural, geographic and financial reasons. However, a small number of CMWs have managed to establish functional practices in the private sector in conservative, infrastructure-challenged rural contexts. With an objective to highlight “what are the successful CMWs doing right given their context?” this paper adopts an asset-based approach to explore the experiences of the Pakistani CMWs who have managed to overcome the barriers and practice. We drew upon ethnographic data that was collected as part of a larger mixed methods study conducted in 2011–2012 in districts Jhelum and Layyah, Pakistan. Thirty eight CMWs, 45 other health care providers, 20 policymakers, 78 women, 35 husbands and 23 older women were interviewed. CMW clinics and practices were observed. Our data showed that only eight 8 out of 38 CMWs sampled were active providers. Poverty as a push factor to work and intrinsic individual-level characteristics that enabled the CMWs to respond successfully to the demands of the midwifery profession in the private sector emerged as the two key themes. Household poverty pushed the CMWs to work in this perceived low-status occupation. Their families supported them since they became the breadwinners. The successful CMWs also had an intrinsic sense of what was required to establish a private practice; they exhibited professionalism, had strong business sense and provided respectful maternity care. The study provides insight into how the program might improve its functioning by adapting its recruitment criteria to ensure selection of right candidates.

## Introduction

Maternal mortality remains a critical public health challenge in most low-income countries, despite three decades of targeted efforts. While awareness and global commitment to safe motherhood has increased, recent data show that overall progress has been slow and countries with the highest burden have failed to achieve their Millennium Development Goal 5 targets. Only 19 out of 183 countries are on track to achieve their target to reduce maternal deaths by three quarters between 1990 and 2015 [1].

Pakistan is one country that had made some progress, but has failed to achieve its MDG-5 target of reducing the maternal mortality rate to 128/ 100,000 live births by 2015 [2]. One postulated reason for this failure is the persistently low rate of skilled birth attendance, particularly in rural areas. Skilled birth attendance is an acknowledged strategy that is key to safe childbirth [3]. However, according to DHS 2012–2013, only 44% of births in rural areas in Pakistan were attended by skilled birth attendants [4].

To increase the proportion of births attended by a skilled provider, the Government of Pakistan introduced a new cadre of rural midwives, the Community Midwives (CMWs), in 2006 [5]. Rural women with ten years of education were recruited and provided 18 months of midwifery training. They were then deployed back to their home villages and expected to establish private practices and provide domiciliary maternity care to a population of 10,000, in geographically defined catchment areas [5]. To-date, 8,000 midwives have been trained, most of whom have returned to their home villages.

Recent evidence suggests, however, that the CMWs have yet to emerge as significant providers. A survey of 1,457 women from districts Layyah and Jhelum, Punjab showed that only 3% and 11.7%, respectively, reported their births were attended by a CMW [6]. Another survey of 2,216 women in the three Baluchistan districts of Lasbela, Gwadar and Ziarat showed that only 0.0%, 0.4% and 0.7% births, respectively, were attended by a CMW [7].

Preliminary work suggests that a large proportion of CMWs are inactive. Program monitoring data from Punjab shows that only 16% of CMWs were conducting four or more deliveries a month—the minimum number required to maintain skills [8]. An early study in districts Layyah and Jhelum, Punjab found that 30 out of 38 CMWs sampled were not working. Mumtaz et al. highlighted the recruitment of unsuitable and uninterested candidates, lack of community trust in CMWs’ skills, perceived unaffordability, gendered mobility limitations and overall poor program implementation as key reasons for the CMWs’ failure to practice [9].

This is a typical example of a deficit or needs-based approach commonly used in the evaluation of maternal health programs. Such an approach presents a negative view that has, possibly inadvertently, compromised progress by presenting individuals, communities and institutions as being solely defined by “problem behaviours”, or even as problems themselves [10]. This often leaves individuals and communities, including program staff and decision-makers, disempowered and feeling incapable of taking charge over their own and their projects’ success [11,12].

The recent past has witnessed an increasing shift in approach to addressing challenges by identifying, emphasising and building upon existing strengths and assets, both at the individual/community and institutional/programmatic levels [13–15]. Here, an asset refers to any factor that enhances the ability of people, communities and institutions to drive the development process. It includes attributes such knowledge, skills, self-esteem, sense of coherence, social networks, reciprocity, mutual aid and collective efficacy, to name a few [16–19]. An asset-based approach focuses and builds upon the capabilities and capacities that already exist at the individual, community and programmatic levels [20]. It provides program policymakers with concrete, actionable evidence that offers context-specific points of leverage which can be used to address local challenges [21].

Currently, little is known about what factors—individual, community and programmatic—enable women to successfully work as community-based skilled birth attendants, particularly in gender-conservative, infrastructure-challenged rural contexts. Studies to-date have tended to focus on ways to improve health worker performance, exploring the role of supervision, leadership, roles and task definition. [22,23]. Another body of literature has focused on workers’ motivations, including monetary and non-monetary incentives, recognition and training opportunities [22,24,25]. Whereas most of this literature has focused on facility-based workers, a smaller body of literature on retention of community health workers has focused on motivations for taking up the occupation in the first place, identifying personal work-related goals, a sense of altruism, self-efficacy, organisational commitment, peer approval and their incentive to work [26–30]. While this literature offers some insight, little is known about the lived experiences of women who manage to successfully provide private-sector midwifery services in contexts that pose numerous gender and infrastructural barriers. What factors facilitate these exceptional women to establish functioning private-sector practices these challenging contexts? This is important to explore for two reasons: 1) While producing a list of external factors and motivations required for success is helpful, “assets most meaningfully manifest only in the transaction between the individual and their environment” [p.153) [31]. Precisely how do individual attributes—knowledge, competencies and self-confidence, interact with social networks, reciprocity, mutual aid and collective efficacy—to overcome the many barriers to produce a successful community midwife? 2) Midwifery service provision is a challenging service to provide, more so than care provided by general community health workers. Midwifery services require night-time care and travel, often over large geographic areas and population sizes, by young women in insecure rural environments. In South Asia, a social stigmatization of the profession is an additional challenge.

This study explores the experiences of the Pakistani CMWs who have managed to establish functional private-sector practices. Specifically, the objective is to highlight not “what are the unsuccessful CMWs doing wrong” but rather, “what are the successful CMWs doing right given their context?” To our knowledge, there is no application of an asset-based lens applied to understand the factors that facilitate young women’s practice of midwifery in the challenging context of rural Pakistan. It will offer the program useful evidence for refining the CMW recruitment criteria and process, to ensure a more targeted and asset-focused selection of candidates.

## Methods

The data presented in this paper are from a larger mixed-methods study that aimed to assess whether the Pakistani CMWs were providing services to poor, marginalised women in rural Pakistan. Data were collected over a 9-month period in 2011–2012 in districts Jhelum and Layyah, Punjab. These two districts were chosen because they were the first in which the program was launched and have sufficient numbers of CMWs to assess coverage. They also span the range of overall levels of development in Punjab, with Jhelum being a relatively well-developed district and Layyah one of the least developed. Agriculture is the main economic activity in both districts. Educational levels are much higher in Jhelum compared to Layyah at 63.9 and 36.7%, respectively [32,33]. Furthermore, Jhelum has a skilled birth attendance rate of 86% and Layyah 52% [34].

Data for the present manuscript are drawn from the qualitative portion of the above-mentioned study [34]. An institutional ethnography was conducted. This qualitative research method enabled us to piece together a picture of how the conduct of health services delivery is coordinated, in relation to ruling ideas and practices [35]. Data were collected from two groups of respondents: 1) Institutional-level respondents, including CMWs, policymakers, program managers and other maternity health care providers; and 2) Community members, including mothers, their husbands, mothers-in-law, and the CMWs’ family members.

A total of 38 CMWs were randomly selected from lists of CMWs trained and deployed by the Punjab government’s Maternal, Newborn and Child Health program. A total of 91 interviews were conducted with 14 CMWs in Jhelum and 24 in Layyah. Based on an initial interview, CMWs were categorised as ‘functional/somewhat functional’ and ‘non-functional’. A “functional/somewhat functional” CMW was defined as having established a routine of work and attending a minimum of 2 deliveries a month, either as indicated by her or observed and verified by the research team. The CMWs were interviewed again, often two to five times to further explore the reasons for their success or failure. If the CMW had a home clinic, it was observed for availability of equipment, cleanliness and evidence of use. The CMWs were also accompanied and observed if and when they made home visits. These visits allowed us to observe moment-in-time patient-provider interactions in routine antenatal and postnatal visits. In addition, at least one, in some cases two, family members of the selected CMWs were interviewed to understand the role of the family members in the establishment of CMW practices. A total of 15 local dais (traditional birth attendants) and 30 other health care providers (non-physician and physician skilled birth attendants and Lady Health Workers) were interviewed at least once, in some cases twice.

Data were collected by a team of four researchers including AB and ZM (PI). ZM and AB have worked together for over a decade, conducting village ethnographies with the objective to map how gender and class inequities impact women's reproductive health. As politically concerned researchers, these authors collect and analyze data through a feminist-inspired critical lens that aims to map how interests and perspectives are controlled by the powerful. Both ZM and AB speak the local language Potohari (Jhelum), and AB speaks Seraiki (Layyah). Both are familiar with the local socio-cultural context. All data were translated and transcribed verbatim into English by a team of local translators and transcribers. ZM double-checked a random sample of transcripts for accuracy and translation. Any discrepancies with original translation were discussed between ZM and AB until consensus was reached with respect to the meaning.

A database of the transcribed interviews and observation notes was created in Atlas-ti, a qualitative data analysis software program. Using a social constructivist, interpretative approach [36], data were inductively coded. Latent content analysis [37] was done to identify domains and themes. Data analysis was an on-going and iterative process throughout all phases of data collection, as early identification allowed a more rigorous probing of unanticipated concepts and variables in the following formal or informal interviews and focus group discussions [38]. An audit trail was maintained throughout the research process to ensure dependability and confirmability [39]. This was achieved through personal memos and journaling throughout the data collection and analysis [40]. Interpretive accuracy was assessed by triangulation of findings across the four phases, peer debriefing within the research team and other colleagues and respondent validation. Researcher bias was reduced through researcher training, peer discussions and respondent validation.

Written consent was obtained from the community midwives, policymakers, program managers and other maternity health care providers, except the traditional birth attendants. Verbal consent was obtained from community members and traditional birth attendants. This was done because this group has low literacy rates. In this context, signing or finger printing documents scares people as it is associated with theft of land. The research team documented their verbal consent by signing on a witness consent form. Permission to accompany CMWs on home visits was provided by the CMW and the client who lived in the home being visited. All respondents’ names have been changed. Ethics approval, which included the approval of verbal consent, was obtained from the University of Alberta Health Research Ethics Board and the National Bioethics Committee, Pakistan.

## Findings

Overall, our data showed that only 8 out of the 38 CMWs interviewed had practices that could be categorised as “functional/somewhat functional”. Analysis of these CMWs data identified two themes:

1. Poverty as a key external factor pushing women to work. The subthemes within the this included household poverty as the main motivation to work for an income, as a key determinant of family support and an unmarried women’s need to reduce the sense of burden on the household which their presence is believed to create;
2. Internal individual-level characteristics that enabled women to respond successfully to the demands of the profession of midwifery in the private sector. Subthemes within this included meta-skills of professionalism and business and providing respectful maternity care.

### Poverty: the key external factor

#### 1) Poverty as a push-factor to work

Economic poverty emerged as an overriding reason for successful CMWs to make an effort to practice. All, except one CMW, were poor women and household poverty was the key push factor that had forced them to work for cash income. Gender norms in this society situate and glorify women as economic dependents. Working as a midwife was, therefore, not a choice, but the result of a need meeting an opportunity. In most cases, the CMWs economic poverty was a recent phenomena—their previous non-poor status best indicated in their receipt of a reasonable education and social connections, two criteria required for entrance into the program. However, unforeseen events such as the death of the father, loss of the husband's income, or more commonly, a non-providing husband necessitated their work. Madiha started working as a CMW after the death of her father in order to support her younger sisters. Noshaba and Kokab needed to work as their husbands' were poor providers.

Also the job 'wandering house to house, day and night' for a woman is only done when the woman is desperate enough to make money

(Field notes, 18th Jan 2012).

Childbirth care in rural Punjab, as in much of South Asia, is understood as polluting, and traditionally, women who performed such work are from low castes and are poor. Such women are perceived to be ritually polluted and therefore suited to undertaking the task. The fact that these CMWs were willing to work in this stigmatized occupation is also indicative of their poverty.

In fact, so powerful was the poverty-pushed need to establish private practices that in some cases, these CMWs put themselves in potentially dangerous situations—as in the case of Kokab, who in the middle of the night was picked up by two unknown men. They claimed to have come from a nearby *katchi abadi* (nomadic tent settlement) where a woman needed childbirth care. While nothing untoward happened, it was only later that Kokab was made to realize that she had put her security in danger to attend a delivery at night. The CMWs working and earning cash income has contributed to alleviating their poverty. In a number of cases they are the primary bread-winners.

#### 2) Poverty-pushed family support

All the successful CMWs had, without exception, support from family members to work as midwives. Family support to work, especially in an occupation requiring door-door travel, often at night, is crucial for CMWs to practice. However, this support is traditionally not forthcoming because gender ideals situate women as economic dependents. Working for cash income, and that too in a stigmatized occupation, is a violation of the gender order that reflects poorly on family men, for it indicates their failure to provide. However, household poverty compelled family members—fathers, mothers and husbands—to support their daughters and wives. Family support included a general permission to work, to travel to people's homes to provide domiciliary care and interact with non-kin members, all of which contravened gender and class norms. The mothers chaperoned the CMWs at all times and the husbands/fathers accompanied them at night.

He does not do any work, so I told him that at the least you can take me around. I have bought him a motorcycle and he drives me around.

(CMW Noshaba).

#### 3) Household burden and unmarried status

Single women were predominant in in our sample of successful midwives, an unusual ratio given that nationally over 95% of women aged 15–49 are married [4]. Although they were poor, an additional push-factor to work was a sense that they were a ‘burden’ to their families. An unmarried woman is viewed as a ‘burden’, first on the aging father and then on the brothers. By working for an income and contributing to household resources, the single women felt their family’s burden was somewhat reduced.

Sadaf was about 32 years old, unmarried and living with her old parents and a brother. Although the family was not poor, Sadaf felt herself to be a burden that might be exacerbated after the parents die. Earning an income as a CMW was one way, Sadaf felt, alleviated the burden.

(Field notes, May 15th 2012).

### Successful response to midwifery professional demands: intrinsic individual characteristics

Two interlinked subthemes identified CMW individual-level characteristics that enabled them to successfully respond to the demands of the midwifery profession, particularly in the private sector.

#### 1) Intrinsic professionalism and business sense

An important facilitator of CMW success was their innate sense of professionalism and business skills. Professionalism is a meta-skill, comprised of situational awareness and contextual judgement that goes beyond technical competencies. It allows individuals to draw on the communication, technical and practical skills appropriate for the given professional scenario [41]. Professionalism in private midwifery practice in this rural Pakistani context included taking initiative, being organized planners, reliable providers and putting in effort to provide the best possible care. For example, one CMW took the initiative to meet the community to inform them of her services. Another requested the District Health Office to accompany a Lady Health Worker on her polio day rounds, for a structured opportunity to enter people's homes and identify pregnant women. Reliability was exemplified when a CMW scheduled her work and then made sure she was available when expected or needed, especially for a night-time birth. For example, Maria had set aside Saturdays to provide antenatal care in a neighbouring village regardless of whether patients were scheduled or not. She also made transport and escort arrangements beforehand to attend an expected birth.

People ask us whether we will be able to come to their homes at night for delivery. We always tell them that no matter what time it is we will come over. (CMW Sabeen)

The goal of coming here every Sunday is not that I have worked lined up or whether I’ll make money on Sundays. There is not work every Sunday here but I come anyways. (CMW Nimra)

Another characteristic of successful CMWs was having an intrinsic understanding of what is required to establish a private practice, or in other words, they possessed business skills. The program expects every CMW to set aside a room in their house as a “home clinic”, and even provided CMWs with some basic equipment in Jhelum. But these successful CMWs went beyond this and either rented or built a clinic on nearby main roads or markets, to increase their visibility. Another CMW had set up a clinic in another village, but within her catchment area that did not have any provider. Nimra printed brochures to advertise her services and even attempted to advertise on local cable TV.

Successful CMWs tended to develop collaborative relationships with other established maternal health care providers such as Lady Health Visitors, nurses, Lady Health Workers, other CMWs and even *dais* (traditional birth attendants), drawing upon their skills and networks to support the establishment of their CMW practices. Partnerships with Lady Health Workers, a type of community health worker, enabled CMWs to identify pregnant women, provide targeted antenatal home visits and garner clients. Partnerships with *dais* enabled CMWs to leverage the community’s trust and provide more holistic midwifery services. Most of these partners tended to see the CMWs as hostile competition, but the CMWs addressed the hostility, for example, by negotiating fee-sharing contracts with their various partners.

Two of the three dais have cooperated with me…one has made me like her daughter and the other is my aunt. I call them for advice during deliveries and they also call me if there are any complicating risk factors. After a delivery I come for a post-natal checkup after a week. The dai’s provide a lot of postnatal care…which is why it is important to keep them as good connections. They do the massages. As soon as I arrived in this area I connected with the dai as she had been working here for many years. Teaming up with them creates a smooth flow. (CMW Nimra)

In general, all successful CMWs had respect for and took pride in midwifery practice in this context, where, as discussed above, delivering babies is a stigmatized occupation.

#### 2) Intrinsic provision of respectful maternity care

The successful CMWs appear to be providing respectful maternity services in a context where such practices are not part of mainstream biomedical training. This includes clearly communicating with their patients, explaining to them at each stage what is happening to their bodies, describing the procedures that are being carried out, why they are needed and being respectful of women's beliefs and practices. For example, Nimra shared with us details of how she ensures that she provided labouring women, whom she referred to a facility, details of what they may expect during childbirth in a facility. Maria reported that she sits with the labouring women throughout their labour, reading to them and massaging to help them through the birth pains. Community women reported that Nimra “was very co-operative and worked with a lot of love and good manners”.

They have so much fear of instruments in their hearts that as soon as [the doctors] take them out the women ask ‘what will they do with that?’ I tell them that it is not always the case that they will do an operation with these tools. These instruments are also used when the placenta comes out. So I satisfy them. When a pregnant woman comes to me I brief her about all of this. (CMW Kaneez)

They also put the amulet on the baby as soon as it was delivered. They asked me to move my hand so that they could put the amulet on that they had purchased from peer sahib…they didn’t even wait for the baby to be bathed…we have to work according to their thinking as well. We give some and we take some. I get some ideas accepted and I accept some of theirs. (CMW Nimra)

## Discussion

The findings presented in this paper highlight the complexity of the external and internal assets that interplay to facilitate establishment of a successful CMW practice in rural Pakistan. These include factors over which the CMWs had little control, like household poverty and the subsequent need to earn income and family support, and being an unmarried, older woman. Factors over which they had control included a sense of professionalism, business skills and provision of respectful maternity care.

The role of household poverty as a key push factor for women to enter the workforce in Pakistan, and in South Asia generally, is well documented [42]. Our research adds to this literature by showing that it is also true of CMWs working as maternal health care providers in rural Pakistan. It has however, added nuance to this evidence base by showing that family support—a key determinant of women’s ability to work in this context—is also determined by this same household poverty.

This leads us to consider the possibility and implications of viewing poverty as an “asset”. Can and should women’s poverty be used as an asset in the achievement of programmatic goals? “Preying” on the economic hardships of women, their families or the community, to perform work that is perceived as unsavoury is a matter for ethical debate. This debate, while occurring in Western countries often in the context of recruitment for militaries [43–45], is nowhere more discussed in South Asia than in the ready-made garment industry in Bangladesh. On one hand, women receive low wages of 68 dollars per month [46] to work 6 days per week, often 10 hour-plus days, in unsafe and unregulated conditions. These women often face sexual harassment and abuse both during their commutes to and from work, as well as in the workplace [47,48]. On the other hand, employment in this industry offers women an income, which in turn has been documented to have positive impacts in their lives, such as postponed age of marriage and childbearing [47].

Logically, if household economic poverty pushes CMWs to work, then recruiting women from poorer households could potentially improve overall program performance, as the workforce would be stocked with women who are, in a way, desperate to earn money to support their households. At first glance, the argument of whether this would be exploitative is the same as the above debate around the garment industry, as it enables an income-generating opportunity via a stigmatized occupation. On the other hand, it can be argued that the CMW program is providing women with 10-years of poor quality education an opportunity to earn an income in remote rural areas—a context bereft of alternative income-generating opportunities. This situation is somewhat different from the Bangladeshi garment workers as these CMWs may be cash-poor, but as our data shows, are not socially poor. Women’s education in Pakistan is a class-related phenomena. Only households that can afford the opportunity costs, both social and financial, of girls’ education will educate their daughters. All CMWs belonged to higher social-economic classes and their poverty was a recent phenomenon [9]. They are independent entrepreneurs with home-based practices and no immediate supervisors. This minimizes exploitation. CMWs may not be as vulnerable to abuse as the ultra-poor, Bangladeshi garment industry workers. In fact, it is highly possible that these higher caste, educated women attending births will elevate the status of the occupation.

Another important characteristic of successful CMWs was that they were more often unmarried, slightly older and wanting to work, so as to be contributors to the household and not be a lifetime burden for their parents. This is a new finding in Pakistan and is not documented elsewhere. At the time of fieldwork, a change in recruitment criteria was under consideration to select only married women. However, our findings suggest this approach may be counterproductive and that a “single” marital status may in fact be an “asset” for the program. Recruiting single, slightly older women who are less likely to get married, are more likely to work, will help the program achieve its objectives of universal skilled birth attendance. It is also beneficial for the CMW, for it enables these women to earn an income in rural Pakistan.

Another asset facilitating successful practice identified is respectful maternity care. Bowser and Hill,[49] in a landscape review of the literature, found disrespect and abuse to be widespread amongst maternity health care providers in developing countries [50–52]. There is evidence to suggest that women who experience abuse and disrespect are both less likely to return to a facility and less likely to suggest facility care for anyone else [52–55]. Disrespectful, abusive behaviour towards patients, particularly poor patients, is normalized and embedded in the culture of clinical practice in Pakistan [49]. Previous research has shown that most unsuccessful CMWs, as members of the same institution, had adopted these practices [34] much to their detriment, since building a clientele in private practice depends on providing client-centred care.

The question that arises then is, “what led these CMWs to provide respectful care and act professionally, given their training did not emphasise such practices?” Can such behaviours be taught, or is it really the case that the sole motivation to earn a profit can lead one to be an efficient, reliable and caring health care provider? While these are subjects of future research, the concept of efficient, reliable and respectful maternity care as an important dimension of quality should be highlighted by health care policymakers at the highest level and incorporated into CMW training.

It is important to note the potential limitations of our study. The findings are based on a large and rich body of qualitative data. Nevertheless, because the study was conducted in two purposively selected districts, questions of generalizability to the national and even provincial level arise. Moreover, only eight midwives provided data that constituted the body of the findings. This number is small, but it is reflective of the reality, of both the challenges women face in this context and of the small size of the assets available. Another limitation could be the role of power dynamics that could have been in play between the researchers and respondents and the potential social-desirability bias that may have resulted. Although the researchers introduced themselves as ‘working for a University’, most respondents, both the community midwives and community women assumed they also reported to the government. This may have led the women to overstate the positive characteristics of their community midwives or the midwives to align their practices with programmatic expectations. While entirely possible, our findings are plausible and our prolonged presence in the field and observations confirmed the respondents’ portrayals of their world. Importantly, data triangulation (interviews, focus group discussions and observations) helped to strengthen the trustworthiness of the data.

To summarise, this research took an asset-based perspective to illuminate the unique characteristics of the few women who had achieved success in establishing a midwifery practice in rural Pakistan. In doing so, we have identified elements that could be used to establish a more targeted recruitment criteria and process. We suggest that the age of the recruits be increased to 27 years and older. By this age, women are generally settled with husbands in their marital village. Their need to work may also become evident by that time. Those not married by this age may feel the need to contribute to their natal households. They are also likely to be more mature and able to handle socially-sensitive reproductive health issues. Furthermore, the recruitment criteria should be adapted to include characteristics of professionalism and work ethic, in addition to academic qualifications. The use of personality tests have been recommended [56] to target specific personality types that may be more conducive to successful outcomes [57]. Lastly, it is imperative to assess applicants’ level of financial need during the interview process.

Through the careful selection of future CMW programme recruits—using intensive recruitment processes focused on selecting the most appropriate candidates—the program will more effectively achieve its outcomes, and rural women will have access to safe childbirths.
